# The complete mitochondrial genome of the wood tiger moth (*Arctia plantaginis*) and phylogenetic analyses within Arctiinae

**DOI:** 10.1080/23802359.2021.1945965

**Published:** 2021-07-05

**Authors:** Juan A. Galarza, Johanna Mappes

**Affiliations:** a Department of Biological and Environmental Sciences, University of Jyväskylä, Jyväskylä, Finland; b Organismal and Evolutionary Biology Research Program, Faculty of Biological and Environmental Sciences, Helsinki University Finland, Helsinki, Finland

**Keywords:** Colour polymorphism, evolution

## Abstract

We report the assembly and annotation of the complete mitochondrial genome of the warningly-coloured wood tiger moth (*Arctia plantaginis*) and investigate its phylogenetic position within Arctiinae. The *A.plantaginis* mitogenome is 15,479 bp long with 13 protein-coding genes, 22 transfer RNAs, 2 ribosomal RNA genes, and an A + T-rich region (D-loop). The phylogenetic analyses based on 13 protein-coding genes showed *A.plantaginis* clustering within a clade of species with white wings and yellow or red bodies. This result can be useful in understanding the evolution of coloration in Arctiid moths.

The wood tiger moth (*Arctia plantaginis*) is a warningly-coloured diurnal species of the Erebidae family widely distributed throughout the Holarctic. In Europe, males are polymorphic having yellow or white hindwings (Hegna et al. 2015). The species is a well-established insect model system in studying the evolution of warning coloration (Stevens and Ruxton 2012). Previous research has relied on partial mitochondrial gene sequences (Galarza et al. 2014), however the complete mitochondrial genome has not been reported.

In this study, we sequenced and annotated the complete mitochondrial genome of *A. plantaginis*. Male adult moths were collected in the surroundings of the city of Jyväskylä, Central Finland (62°19′19.2″N, 25°48′55.1″E) during flying season in June 2019. The samples were dry-frozen at −20 °C and are preserved at the Dpt. of Biological and Environmental Science of the University of Jyväskylä with voucher ID CFI-19-010; contact Dr. Juan Galarza (juan.galarza@jyu.fi). The samples were sequenced in an Illumina MiSeq (2 x300bp PE). Mitochondrial reads were then identified by mapping high-quality reads to mitogenomes of other Arctiids using bowtie2 (Langmead and Salzberg 2012) and extracted using SAMtools (Li et al. 2009). The mitogenome assembly and annotation was carried out using MitoZ v2.4-alpha (Meng et al. 2019).

The *A. plantaginis* mitochondrial genome is circular in shape with a total length of 15,479 bp consisting of 13 protein-coding genes, 22 tRNA, 2 rRNA genes, and an A + T-rich region (D-loop). The major strand encodes 9 protein-coding genes and 14 tRNAs, whereas than the minor strand encodes 4 protein-coding genes, 2 rRNA genes, and 8 tRNAs. The two rRNAs (16S rRNA and 12S rRNA) are separated by tRNA-Val and are located between tRNA-Leu and the A + T-rich region. The composition and arrangement of the mitogenome is comparable to other diurnal Erebidae (Lu et al. 2013).

The phylogenetic position of *A.plantaginis* was investigated through a phylogenetic tree including eleven Arctiinae mitogenomes from *Eilema ussuricum* (Daniel, 1954) GenBank Accession: MN696172, *Vamuna virilis* (Rothschild, 1913) GenBank Accession: KJ364659, *Paraona staudingeri* (Alphéraky, 1897) GenBank Accession: KY827330.1, *Callimorpha dominula* (Linnaeus, 1758) GenBank Accession: KP973953.1, *Aglaomorpha histrio* (Walker, 1855) GenBank Accession: KY800518.1, *Hyphantria cunea* (Drury, 1773) GenBank Accession: NC_014058.1, *Spilosoma lubricipeda* (Linnaeus, 1758) GenBank Accession: NC_050385.1, *Lemyra melli* (Daniel, 1943) GenBank Accession: KP307017.1, *Spilarctia subcarnea* (Walker, 1855) GenBank Accession: KT258909.1, *Arctia plantaginis* (Linnaeus, 1758), *Nyctemera arctata* (Walker, 1856) GenBank Accession: KM244681.1, *Amata formosae* (Butler, 1876) GenBank Accession: NC_021416.1, and one Lymantriidae *Somena scintillans* (Walker, 1856) GenBank Accession: MH051839 as an outgroup. The protein-coding genes of all species were aligned using MUSCLE v3.8.425 (Edgar 2004) and a maximum likelihood phylogenetic tree was constructed using RAxML (Stamatakis 2014). The resulting phylogeny (Figure 1) identified *A.plantaginis* clustering in its own branch within a clade including *Spilarctica subcarnea*, *Leymra melli*, *Hyphantria cunea*, and *Siplosoma lubricipeda*. Interestingly, all species in this clade, with the exception of *A.plantaginis,* have white wings, although they all display a yellow or red body. This result can be useful in understanding the evolution of warning coloration in Arctiid moths.

**Figure 1.**
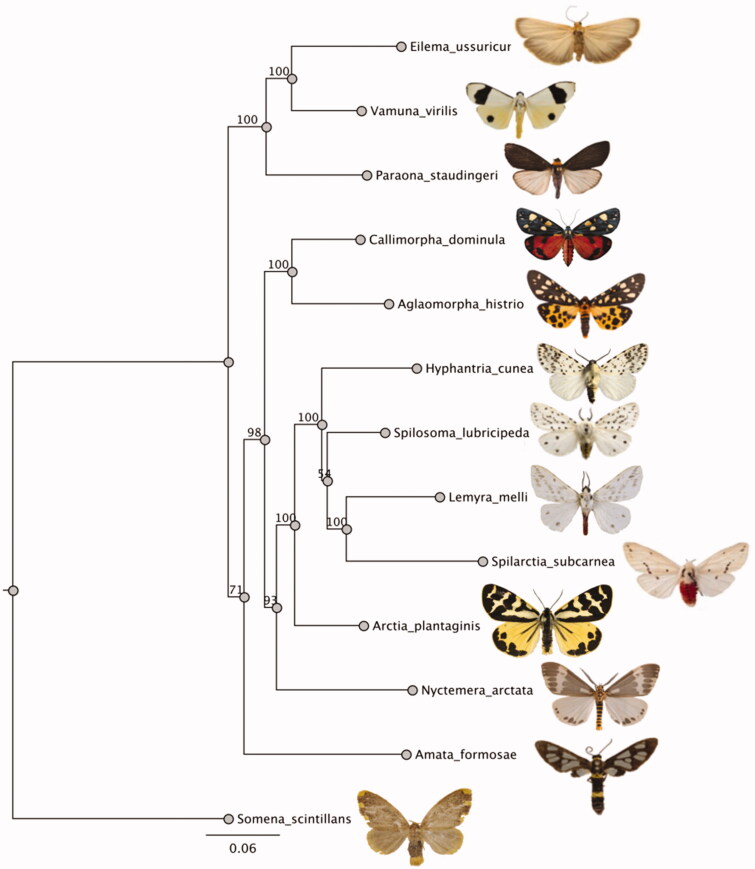
Maximum-likelihood tree of evolutionary relationships of *Arctia plantaginis* and 11 Arctiid moths based on all coding-protein genes of complete mitogenomes. Bootstrap support values are indicated next to nodes. *Somena scintillans* (Lymantriidae) was used as an outgroup.

## Data Availability

The genome sequence data that support the findings are freely available in GenBank of NCBI (https://www.ncbi.nlm.nih.gov) under accession number MW394229. The raw sequence data is deposited in SRA of NCBI with accession SRP323199, BioProject PRJNA735917. Credit for photographs used in the tree: *Eilema ussuricum*: https://en.wikipedia.org/wiki/Eilema. *Vamuna virilis*: Hsu Hong Lin - Flickr: A09-20091016-P064, CC BY-SA 2.0, https://commons.wikimedia.org/w/index.php?curid=17246537. *Paraona staudingeri:* https://en.wikipedia.org/wiki/Paraona_staudingeri. *Callimorpha dominula:* http://perhoset.perhostutkijainseura.fi/historia/arctiinae/cal-dominula.htm. *Aglaomorpha histrio:* Hsu Hong Lin - Flickr: A01-20090819-P020, CC BY-SA 2.0, https://commons.wikimedia.org/w/index.php?curid=17268458. *Hyphantria cunea:* https://commons.wikimedia.org/wiki/File. *:Hyphantria_cunea_male.JPG* *Spilosoma lubricipeda:* M. Virtala https://commons.wikimedia.org/w/index.php?curid=11921337. *Lemyra melli:* http://www.jpmoth.org/∼dmoth/Digital_Moths_of_Asia/90_NOCTUOIDEA/02_EREBIDAE/02_ARCTIINAE/03_Arctiinae/02_Lemyra/Lemyra%20melli/Lemyra%20melli.htm. *Spilarctia subcarnea:* http://szmn.eco.nsc.ru/Lepidop/images/Spilarctia_subcarneum.htm. *Arctia plantaginis*: http://perhoset.perhostutkijainseura.fi/historia/arctiinae/par-plantaginis.htm. *Nyctemera arctata:* https://commons.wikimedia.org/w/index.php?curid=17432567. *Amata formosae:* https://catalog.digitalarchives.tw/item/00/65/77/5e.html. *Somena scintillans:* http://twmoth.tesri.gov.tw/peo/MothInfo/A58-20180610-041.
